# Do initial hematologic indices predict the severity of COVID-19 patients?

**DOI:** 10.3906/sag-2007-97

**Published:** 2021-02-26

**Authors:** Ali ASAN, Yasemin ÜSTÜNDAĞ, Nizameddin KOCA, Abdullah ŞİMŞEK, Halil Erkan SAYAN, Hülya PARILDAR, Burcu Dalyan CİLO, Kağan HUYSAL

**Affiliations:** 1 Department of Infectious Diseases and Clinical Microbiology, University of Health Sciences, Bursa Yüksek İhtisas Training and Research Hospital, Bursa Turkey; 2 Department of Clinical Biochemistry, University of Health Sciences, Bursa Yüksek İhtisas Training and Research Hospital, Bursa Turkey; 3 Department of Internal Medicine, University of Health Sciences, Bursa Yüksek İhtisas Training and Research Hospital, Bursa Turkey; 4 Department of Pulmonary Diseases, University of Health Sciences, Bursa Yüksek İhtisas Training and Research Hospital, Bursa Turkey; 5 Department of Anesthesiology and Reanimation, University of Health Sciences, Bursa Yüksek İhtisas Training and Research Hospital, Bursa Turkey; 6 Department of Family Medicine, University of Health Sciences, Tepecik Trainingand Research Hospital , İzmir Turkey; 7 Department of Microbiology, University of Health Sciences, Bursa Yüksek İhtisas Training and Research Hospital, Bursa Turkey

**Keywords:** Intensive care unit, COVID-19, neutrophils, leukocytes, platelets

## Abstract

**Background/aim:**

In this study, we aimed to evaluate the initial hematological findings analyzed on admission in confirmed COVID-19 patients who were transferred to the intensive care unit (ICU), to predict possible hematological indices.

**Materials and methods:**

Initial neutrophil to lymphocyte ratio (NLR), platelet to lymphocyte ratio (PLR), monocyte to lymphocyte ratio (MLR), red cell distribution width to platelet ratio (RPR), mean platelet volume to platelet ratio, and lymphocyte multiplied by platelet count (LYM × PLT), of 695 patients with laboratory-confirmed COVID-19 were investigated and comparisons were made between the mild/moderate and severe groups.

**Results:**

The proportion of COVID-19 cases admitted to the ICU was 3.9%. The median age of patients admitted to the ICU was significantly higher than those who were not; [68.5 (interquartile range (IQR); 21.5] years vs. 41.0 (IQR; 15.7) years; P < 0.001.Severe cases had higher NLR (6.6 vs. 2.4; P < 0.001), and MLR (0.40 vs. 0.28; P = 0.004) and lower PLR (180.0 vs. 129.0; P < 0.001) compared to that of mild or moderate patients. Among all of the parameters, the ROC curve of NLR gave us the best ability to distinguish serious patients at an early stage (AUC = 0. 819, 95% confidence interval 0.729–0.910; P < 0.001).

**Conclusion:**

These data showed that age, initial NLR, PLR, and LYM × PLT were associated with the severity of COVID-19 disease and patients’ need for the ICU. Therefore, initial hemogram parameters may be essential to predict the prognosis of COVID-19 patients.

## 1. Introduction

In December 2019, a novel coronavirus, severe acute respiratory syndrome coronavirus 2 (SARS-CoV-2), was identified in Wuhan, China; and quickly spread around the World [1]. The World Health Organization named this infection COVID-19 and announced a pandemic1WHO (2020). WHO: Novel coronavirus (2019-nCoV) situation report-51 2020 [online]. Website https://www.who.int/docs/default-source/coronaviruse/situation-reports/20200311-sitrep-51-covid-19.pdf?2 [accessed 10 May 2020].. In our country, the first PCR positive COVID-19 case was detected on March 11th, 20202Republic of Turkey Ministry of Health (2020). COVID-19 Information Page [online]. Website https://covid19.saglik.gov.tr/ [accessed 14 May 2020]..

The clinical spectrum of COVID-19 infection can vary from asymptomatic to severe disease (e.g., acute respiratory distress syndrome [ARDS], acute cardiac injury, and acute kidney injury) [1,2]. Up to 32% of all positive patients require intensive care unit (ICU) admission, and death can occur [2,3]. Therefore, early identification of patients with severe disease risk and those that may potentially develop a life-threatening condition is important.

Increasing evidence supports the role of a dysfunctional immune response to the airway damage and the progression of various viral pneumonia, including COVID-19 [4,5]. Hematological indices, such as neutrophil to lymphocyte ratio (NLR), platelet to lymphocyte (PLR) ratio, monocyte to lymphocyte ratio (MLR), lymphocyte count multiplied by platelet count (LYM × PLT), red cell distribution width to platelet ratio (RPR), are indicators of the systemic inflammatory response and have been extensively investigated in various diseases [6–9]. Abnormal blood count results were detected in patients with COVID-19 [4,10–12], which are considered to be potential predictors of the outcomes [13,14]. 

In this study, we aimed to identify hematological indices of confirmed COVID-19 patients that were analyzed on admission and their relationship with the disease severity, in order for them to be used in predicting the disease progression. 

## 2. Materials and methods

The study was approved by the ethics committee of SBU Bursa Yüksek İhtisas Training and Research Hospital (No. 2020/05-02); a pandemic hospital with 1430 beds served as the setting for the present retrospective cohort study. The study was conducted in accordance with the principles outlined in the Declaration of Helsinki.

Combined nasopharyngeal and oropharyngeal swabs were taken from 1100 patients who met the suspected case definitions for COVID-19. Real-time quantitative amplification of SARS CoV RNA was performed with SARS-CoV-2 (2019-nCoV) RT-qPCR Detection Kit (Bio-Speedy®, Bioeksen R&D Technologies, İstanbul, Turkey) with a thermal cycler device (CFX96TM Real-Time System, Bio-Rad Laboratories, Singapore) according to the manufacturer’s instructions. Six-hundred and ninety-five confirmed SARS-CoV-2 patients admitted to our hospital between March 27th and April 30th, 2020, were included in the study. Individuals younger than 18 years old, those who required aggressive treatment within 24 h of admission, and those with an absence of initial complete blood count (CBC) test results were excluded from the study.

Twenty-seven patients who fulfilled one of the following criteria; dyspnea and respiratory distress; respiratory rate > 30/min, PaO2 / FiO2 <300; SPO2 <90 despite 5 L/min oxygen treatment, PaO2 <70, hypotension (systolic blood pressure <90 mmHg and/or more than 40 mmHg decrease and mean arterial pressure <65 mmHg), tachycardia >100/min, acute kidney injury, development of acute organ dysfunction, patients with immunosuppression, acute bleeding diathesis, troponin increase, arrhythmia, lactate > 2 mmol, capillary return disorder, and cutis marmaratus were transferred to ICU. 

The initial triage protocol of COVID-19 patients involves drawing blood samples upon admission. CBCs were analyzed within 1 h after venipuncture using an automatic blood counter (Mindray BC-5800; Mindray Bio-medical Electronics Co. Ltd., Shenzhen, China). Mean platelet volume divided by platelet count (MPV to PLT) and lymphocyte multiplied by platelet (LYM × PLT) were recorded. NLR, PLR, and MLR were calculated by dividing the number of neutrophils, platelet, and monocyte count by the lymphocyte count. RDW to platelet ratio (RPR) was calculated using the formula (RDW ×100 / PLT (10^9^ / L) [15].

## 3. Statistical analysis

Statistical analysis was performed using SPSS (Statistical Package for Social Sciences) for Windows (SPSS Inc, Chicago, IL), and P values of <0.05 were considered significant. Descriptive statistics are presented as frequency distribution and percentage. The Kolmogorov–Smirnov test was used to identify the distribution of variables. Statistically significant differences between the variables were established using the Mann­Whitney U test. A binary logistic regression analysis was performed to determine the influence of age and all other significant factors.

## 4. Results

The proportion of COVID-19 cases admitted to ICU was 3.9%. Signs and symptoms of severe cases on admission included self-reported fever (52%), cough (44%), shortness of breath (52%), sore throat (19%), myalgia/arthralgia (19%), fatigue (15%), chest pain/discomfort (11%), nasal symptoms (11%), headache (11%), nausea/vomiting (11%) and diarrhea (4%).

During the study period, the median age of patients who were transferred to ICU was significantly higher than those who were not transferred [68.5 (interquartile range (IQR); 21.5) years vs. 41.0 (IQR; 15.7) years; P <0.001, Table 1].

**Table 1:** Initial laboratory findings of patients with COVID-19.

	Group 1 (N = 668)	Group 2 (N = 27)	P value
Age, median (IQR)	41.0 (15.7)	69.0 (21.0)	<0.001
Male/female (%)	47.3/52.7	55.6/44.4	
WBC count, ×109/L	6.2 (2.95)	8.3 (5.5)	0.008
Haemoglobin, g/dL	13.8 (2.3)	12.9 (4.0)	0.012
Hct (%)	40.8 (5.9)	38.5 (10.3)	0.017
Platelet count, × 109/L	217 (74)	192 (90)	0.081
NEU (%)	63.8 (16.7)	82.7 (21.5)	<0.001
LYM (%)	26.6 (15.7)	12.7 (12.6)	<0.001
MONO (%)	7.6 (4.0)	5.9 (5.0)	0.004
EOS (%)	0.7 (1.3)	0.1(0.3)	<0.001
MCV, mm3	85.7 (5.8)	84.4 (9.0)	0.321
MCH, pg	29.1 (2.5)	28.2 (4.3)	0.391
MCHC (g/dL)	33.8 (1.2)	33.3 (1.3)	0.003
RDW, %	13.1 (1.2)	13.8 (3.1)	0.007
MPV, fL	9.6 (1.3)	9.7 (1.4)	0.241
CRP, mg/L	6.2 (13)	73 (123)	<0.001
MPV to PLT	0.044 (0.018)	0.053 (0.028)	0.049
NLR	2.4 (2.0)	6.6 (7.8)	<0.001
PLR	129 (70)	180 (156)	<0.001
MLR	0.28 (0.2)	0.40 (0.3)	0.004
LYM × PLT	346.5 (274.9)	169.3 (212.7)	<0.001
RPR	0.062 (0.020)	0.076 (0.028)	0.001

The patients were divided into two groups: Group 1: Patients not requiring ICU admission; Group 2: ICU admission required. ICU = intensive care unit; IQR = interquartile range; RBC = red blood cell; WBC = white blood cell; Hct = hematocrit; NEU = neutrophil; LYM = lymphocyte; MONO = monocyte; EOS = eosinophils; BASO = basophile; MCV = mean corpuscular volume; MCH = mean corpuscular haemoglobin, MCHC = mean corpuscular hemoglobin concentration, RDW = red cell distribution width; MPV = the mean platelet volume; NLR = neutrophil to lymphocyte ratio; MLR = monocyte to lymphocyte ratio; LYM × PLT = lymphocyte multiplied by platelet; RPR = red cell distribution width to platelet ratio; MPV to PLT = mean platelet volume to platelet count ratio. Data are median (IQR) or n (%).

When comparing the initial hemoglobin concentration [12.9 (4.0) vs. 13.8 (2.3) g/dL; P < 0.001] and lymphocyte count [12.7% (12.6) vs. 26.6% (15.7)] both values were found to be lower than that of nonsevere cases (Table 1).

It is observed that severe cases had higher NLR (6.6 vs. 2.4; P < 0.001), and MLR (0.40 vs. 0.28; P = 0.004) and lower PLR (180.0 vs. 129.0; P < 0.001) compared to mild or moderate patients (Table 1). The ROC curve of NLR gave us the best prediction opportunity for distinguishing patients with severe disease from an earlier stage (AUC = 0.819, 95% confidence interval 0.729–0.910; P < 0.001, Table 2).

**Table 2:** Diagnostic performances of initial hematologic indices for distinguishing admission to ICU.

Parameter	AUC (95% CI)
NLR	0.819 (0.729–0.910)
LYM × PLT	0.786 (0.706–0.865)
PLR	0.746 (0.629–0.862)
RPR	0.686 (0.584–0.788)
MLR	0.663 (0.560–0.765)
MPV to PLT	0.612 (0.492–0.732)

AUC (95% CI) = area under the receiver operating characteristic curve (95% confidence interval); NLR = neutrophil to lymphocyte ratio; LYM = lymphocyte; PLT = platelet; MPV = mean platelet volume; PLR = platelet to lymphocyte ratio; RPR = red cell distribution width to platelet count; MLR = monocyte to lymphocyte ratio; MPV to PLT = mean platelet volume to platelet count ratio.

Potential risk factors, including age, NLR, PLR, LYM × PLT, and RDW were investigated using binary logistic regression analysis. Age was found to be the only significant (b = 0.070, SE = 0.015, P < 0.001) positive predictor for ICU requirement, with the OR 1.073 (95% CI, 1.042 to 1.104) (Table 3).  

**Table 3:** Binary logistic regression analysis predicting the likelihood of admission to the ICU.

Variable	B	S.E.	OR with 95% Cl	P value
Age	0.070	0.015	1.073 (1.042–1.104)	<0.001
Sex	–0.178	0.479	0.837(0.327–2.141)	0.711
NLR	0.106	0.073	1.112(0.964–1.281)	0.145
LYM × PLT	–0.003	0.002	0.997 (0.994–1.001)	0.209
PLR	0.005	0.003	1.005 (0.999–1.011)	0.086
RPR	2.620	12.244	13.738 (0.00–363400)	0.831
MLR	0.340	0.967	1.405 (0.211–9339)	0.725
MPV to PLT	4.471	12.143	87.422 (0.00–189700)	0.713

OR = odds ratio; Cl = confidence interval; NLR = neutrophil to lymphocyte ratio; LYM = lymphocyte; PLT = platelet; MPV = mean platelet volume; PLR = platelet to lymphocyte ratio; RPR = red cell distribution width to platelet; MLR = monocyte to lymphocyte ratio; MPV to PLT = mean platelet volume to platelet count ratio.

## 5. Discussion

On admission, the COVID-19 patients who needed to be taken to the ICU were older than those that did not require ICU intervention, as reported before [1,2]. In the ICU-patient group, the initial absolute neutrophil count was higher, and the lymphocyte count was lower among patients who were transferred to the ICU compared to mild cases who were not. This is in accordance with the previous publications that reported the increase in leukocyte count and a decrease in lymphocyte count as an indicator for clinical deterioration in COVID patients [16,17].

Neutrophils are the most important cellular defense line against infections, which first respond to viral invasion, and limit the viral replication and spread. However, neutrophils have also been reported to mediate deleterious effects on the host during viral infection [18].

The immune response to a viral infection is mainly based on lymphocytes. It has been assumed that a significant decrease in lymphocyte count may be due to increased lymphocyte consumption, destruction of lymphatic tissues, and cytokine-induced T-cell apoptosis in patients with COVID-19 [13,19]. Lymphopenia, as a sign of the severe disease, is not specific to COVID-19. It has also been seen in other viral causes of pneumonia, such as influenza [20,21].

Notably, we observed that COVID-19 patients who were transferred to the ICU had lower lymphocyte count and higher NLR and PLR. In consistency with our study, Yang et al. suggested NLR ≥3.3 as an indicator with clinical symptoms to change disease status from mild to severe disease [22]. NLR is reported to be used as an early indicator for the severe disease, similar to previous findings from a different patient population [22,23].

In the current study, the median PLR of severe COVID patients was higher compared to that of nonsevere cases. The PLR, which reflects both aggregation and inflammation control, is a better marker for the decision of patients for transfer to the ICU than platelet or lymphocyte count alone. Changes in platelet count and activity are closely related to various diseases [24]. It has been shown in several studies that low platelet count is directly related to the severity of the disease in COVID-19 patients [12,25]. Platelets not only contribute to hemostasis but also participate in the inflammation and host defense. Decreased platelet production and increased consumption due to diffuse alveolar damage are thought to cause thrombocytopenia in COVID-19 patients [26,27].

In accordance with previous studies, the hemoglobin level was found to be lower in COVID patients who needed to be taken to the ICU when compared to that of milder COVID patients [28,29]. RDW is a measure of the change in erythrocyte volume and has been reported to be a predictor of mortality in some conditions, including infections [30]. The higher RDW is associated to increased inflammation markers which have been tightly correlated with the critical disease [31,32].

## 6. Conclusion

According to the results of this study, the variables, including the age of the patients, NLR, PLR, and LYM × PLT, are related to the severity of COVID-19 disease and may contribute to decision making for transferring patients to the ICU on admission. Therefore, initial CBC parameters should be monitored for predicting the prognosis of COVID-19 patients.

Limitation: This study was a retrospective, single-center study. Prospective and multicenter clinical studies might be required to avoid a certain degree of deviation and to support the findings. 

## Informed consent

SBU Bursa Yüksek İhtisas Etik Kurul 2011-KAEK-25 2020/05-02.
